# Association between intraoperative pulmonary artery pressure and cardiovascular complications after off-pump coronary artery bypass surgery: a single-center observational study

**DOI:** 10.1186/s12871-023-02057-5

**Published:** 2023-04-06

**Authors:** Mitsuhiro Matsuo, Toshio Doi, Masahito Katsuki, Yuichiro Yoshimura, Hisakatsu Ito, Kazuaki Fukahara, Naoki Yoshimura, Mitsuaki Yamazaki

**Affiliations:** 1 Department of Anesthesiology, Faculty of Medicine, University of Toyama, 2630 Sugitani, 930-0194 Toyama, Japan; 2 First Department of Surgery, University of Toyama, Toyama, Japan; 3 Department of Neurosurgery, Itoigawa General Hospital, Itoigawa, Japan; 4 School of Engineering, Chukyo University, Nagoya, Japan; 5 Department of Anesthesiology, Toyama Nishi General Hospital, Toyama, Japan

**Keywords:** Artificial intelligence, Postoperative complication, Pulmonary hypertension, Off-pump coronary artery bypass surgery, Time-series clustering

## Abstract

**Background:**

The impact of intraoperative pulmonary hemodynamics on prognosis after off-pump coronary artery bypass (OPCAB) surgery remains unknown. In this study, we examined the association between intraoperative vital signs and the development of major adverse cardiovascular events (MACE) during hospitalization or within 30 days postoperatively.

**Methods:**

This retrospective study analyzed data from a university hospital. The study cohort comprised consecutive patients who underwent isolated OPCAB surgery between November 2013 and July 2021. We calculated the mean and coefficient of variation of vital signs obtained from the intra-arterial catheter, pulmonary artery catheter, and pulse oximeter. The optimal cut-off was defined as the receiver operating characteristic curve (ROC) with the largest Youden index (Youden index = sensitivity + specificity – 1). Multivariate logistic regression analysis ROC curves were used to adjust all baseline characteristics that yielded *P* values of < 0.05.

**Results:**

In total, 508 patients who underwent OPCAB surgery were analyzed. The mean patient age was 70.0 ± 9.7 years, and 399 (79%) were male. There were no patients with confirmed or suspected preoperative pulmonary hypertension. Postoperative MACE occurred in 32 patients (heart failure in 16, ischemic stroke in 16). The mean pulmonary artery pressure (PAP) was significantly higher in patients with than without MACE (19.3 ± 3.0 vs. 16.7 ± 3.4 mmHg, respectively; absolute difference, 2.6 mmHg; 95% confidence interval, 1.5 to 3.8). The area under the ROC curve of PAP for the prediction of MACE was 0.726 (95% confidence interval, 0.645 to 0.808). The optimal mean PAP cut-off was 18.8 mmHg, with a specificity of 75.8% and sensitivity of 62.5% for predicting MACE. After multivariate adjustments, high PAP remained an independent risk factor for MACE.

**Conclusions:**

Our findings provide the first evidence that intraoperative borderline pulmonary hypertension may affect the prognosis of patients undergoing OPCAB surgery. Future large-scale prospective studies are needed to verify the present findings.

**Supplementary Information:**

The online version contains supplementary material available at 10.1186/s12871-023-02057-5.

## Background

Intraoperative hemodynamic perturbations are commonly caused by anesthetic management, surgical interventions, and medical comorbidities. There is increasing evidence that perturbations in routinely obtained vital signs predict the postoperative adverse outcomes. For example, intraoperative hypotension is related to postoperative kidney injury, myocardial injury, and death, depending on the degree and duration of hypotension in non-cardiac surgery [1]. Intraoperative blood pressure variability is also associated with postoperative mortality [2]. Moreover, high arterial oxygen partial pressure during cardiac surgery is reportedly associated with acute kidney injury [3, 4]. However, although the influence of vital signs derived from left heart function on postoperative complications is known, the importance of hemodynamic variables obtained from the pulmonary artery catheter (PAC) remains unknown. We performed a retrospective observational study to evaluate the relationship between intraoperative vital signs, including pulmonary hemodynamics, and postoperative cardiovascular events in patients who underwent off-pump coronary artery bypass (OPCAB) surgery.

## Methods

### Study design and settings

This single-center, retrospective, observational study was performed at Toyama University Hospital, which is an academic, teaching, and tertiary care center that covers a population of 1 million in Japan. This study was approved by the Ethics Committee of Toyama University Hospital, Toyama, Japan (approval no. R2021079) on 7 September 2021, and adhered to the principles of the Declaration of Helsinki. The ethics committee waived the requirement for written informed consent because of the retrospective nature of the study. Instead, opt-out consent documents were presented on our hospital website for patients who did not wish to participate. This study was performed in accordance with the Strengthening the Reporting of Observational studies in Epidemiology guidelines [5].

### Patients

We identified all isolated OPCAB surgeries that were performed at our hospital between November 2013 and July 2021. There was a single adult cardiac surgical team at our hospital during the study period, with one responsible surgeon (K.F.). Patients with incomplete data and those who were converted to on-pump coronary artery bypass surgery were excluded.

### Surgical procedure

OPCAB surgeries were performed during the study period through a median sternotomy as described previously [6]. Briefly, the heart was displaced by applying traction to the pericardium with a supporting thread. An Octopus IV cardiac stabilizer (Medtronic, Minneapolis, MN, USA) was used to fix the coronary artery. A Starfish heart positioner (Medtronic, Minneapolis, MN, USA) was used to expose the circumflex, right coronary, and posterior descending arteries. Heparin sodium (200 units/kg) was administrated intravenously before the arterial conduits were divided. During anastomosis of the anterior descending artery, distal coronary flow was maintained using an external shunt tube, whereas during anastomosis of the circumflex artery or right coronary artery, coronary flow was transiently occluded by simple clamping. After anastomosis, protamine sulfate (2 mg/kg) was administered intravenously.

### Data collection

We retrieved patients’ characteristics, surgical records, and postoperative complications from the electronic medical records. From the anesthesia record (Clinical Network System CAP-2000; Nihon Kohden, Tokyo, Japan), we extracted the data measured every minute after induction of general anesthesia using an intra-arterial catheter, central line, PAC, and pulse oximeter. Vital signs were recorded until the patient left the operating theatre. As mean values, arterial blood pressure of 0 to 200 mmHg, pulmonary artery pressure (PAP) of 0 to 40 mmHg, and central venous pressure (CVP) of − 5 to 25 mmHg were considered for analysis; other values were considered outliers.

### Outcome

The primary outcome of this study was the development of major adverse cardiovascular events (MACE) (a composite of cardiovascular death, non-fatal myocardial infarction, unstable angina, heart failure, stroke, and other cardiovascular events requiring further interventions) during hospitalization or within 30 days after OPCAB surgery [7]. Cardiovascular death was defined as death due to myocardial or cerebral infarction or sudden cardiac death. Myocardial infarction was defined as electrocardiographic changes or elevated troponin concentrations consistent with clinical signs (chest pain or discomfort). Unstable angina was defined based on the Braunwald criteria [8]. Heart failure was defined based on radiographic findings (pulmonary congestion) and the need for further interventions by cardiologists. Stroke was defined as a new focal neurological deficit, including transient ischemic attack. Stroke was further classified as being the result of intracranial hemorrhage, ischemia (if computed tomography or magnetic resonance imaging results were available), or an uncertain cause. Other cardiovascular events include peripheral artery diseases, dissecting aneurysm of the aorta, and increased size of the aortic aneurysm.

### Time-series clustering

Clustering is the division of a set of classification objects into subsets so that internal cohesion and external isolation are achieved, providing insight into the differences in patients’ phenotypes, independent of existing diagnoses (unsupervised learning) [9]. Time-series clustering, one of the clustering methods, is an unsupervised data mining technique for time series data and is employed as a standard tool in data science for anomaly detection, character recognition, pattern discovery, and visualization of time series. In this study, time-series clustering was applied to statistically significant variables using Python 3.9.0 and tslearn 0.5.2 (https://tslearn.readthedocs.io/en/stable/). We performed time-series clustering using dynamic time warping (DTW) because the anesthetic time differed among the individual patients. DTW is a method used to measure the distance and similarity between time-series data. DTW finds the distance (absolute value of the error) between each point of two time-series data by brute force, and finds the path that is the shortest between the two time-series data after comparing all distances. The similarity can be calculated even if the lengths and periods of the time series are different [10]. The number of clusters was determined by considering the highest silhouette scores [11]. The silhouette score is a metric used to calculate the goodness of fit of a clustering technique. Its value ranges from -1 to 1. Silhouette coefficient values near + 1 indicate that the sample is far away from the neighboring clusters. A value of 0 indicates that the sample is on or very close to the decision boundary between two neighboring clusters, and negative values indicate that those samples might have been assigned to the wrong cluster. We determined the number of clusters with the biggest silhouette score.

### Statistical analysis

Given the observational nature of the study, sample size was not calculated a priori. Continuous values are expressed as mean ± standard deviation or median (interquartile range [IQR]). The coefficient of variation was calculated by dividing the standard deviation by the mean. Statistical comparisons were performed using an unpaired two-tailed t-test and the Mann–Whitney U test or Fisher’s exact test. Statistical significance was set at *P* < 0.05. Multivariate analyses adjusted all baseline characteristics and vital signs that yielded *P* values of < 0.05 in the univariate analyses. The optimal cut-off was defined as the receiver operating characteristic (ROC) curve with the largest Youden index (Youden index = sensitivity + specificity – 1). The discrimination performances were assessed with the DeLong test, net reclassification improvement, and integrated discrimination improvement. All statistical analyses were performed using EZR, which is a graphical user interface for R (The R Foundation for Statistical Computing, Vienna, Austria) [12].

## Results

### Patient and surgical characteristics

**Fig. 1:**
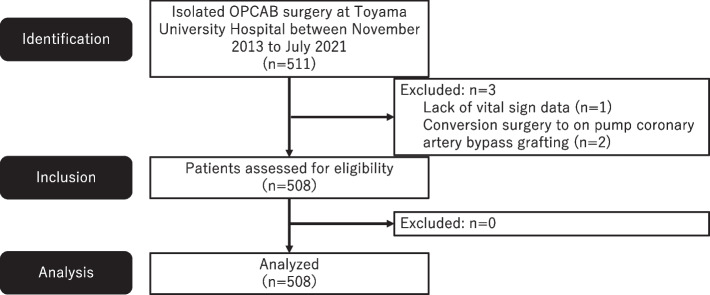
Flow chart of study enrolment

In total, 511 isolated OPCAB surgeries were performed during the study period (Fig. 1). After excluding one patient with incomplete vital sign data and two patients who were converted to on-pump coronary artery bypass surgery, we analyzed the remaining 508 patients (Table 1). The mean patient age was 70.0 ± 9.7 years, and 399 (79%) were male. The mean preoperative left ventricular (LV) ejection fraction (LVEF) was 57.5 ± 12.7%, and the median New York Heart Association (NYHA) cardiac function classification was 2 (IQR, 1–2). OPCAB involved 3.0 ± 1.0 vessel anastomoses, an operative time of 208 ± 47 min, and blood loss of 908 mL (IQR, 619–1266). There were three patients with rheumatoid arthritis without interstitial pneumonia, but no patients with other connective tissue diseases. In all patients, anesthesia was induced with midazolam and maintained with sevoflurane after intubation. A PAC was inserted after induction of anesthesia in all OPCAB procedures.

**Table 1:** Preoperative patient demographics and surgical characteristics

Patients	
Age, years	70.0 ± 9.7
Sex, male	399 (79)
Smoking history	313 (62)
BMI, kg/m^2^	23.7 ± 3.3
NYHA functional class	2 (1–2)
Hypertension	418 (82)
Dyslipidemia	403 (79)
Diabetes	307 (60)
Stroke	103 (60)
Atrial fibrillation	43 (8)
Malignancy	12 (2)
Hemodialysis	50 (10)
Pulmonary hypertension	0 (0)
Preoperative beta blocker use	314 (62)
Preoperative statin use	350 (69)
Hemoglobin, g/dL	11.5 ± 1.9
LVEF, %	57.5 ± 12.7
Moderate/severe MR	15 (3)
Estimated GFR, mL/min/1.73 m^2^	57.7 ± 25.6
Pacemaker implanted	12 (2)
Scheduled IABP use	221 (44)
Surgery	
Operative time, min	208 ± 47
Intraoperative blood loss, mL	908 (619–1266)
Number of anastomoses	3.0 ± 1.0
LAD + diagonal branch	1.4 ± 0.5
LCX	0.9 ± 0.6
RCA	0.7 ± 0.6
Emergency surgery	20 (4)

Data are shown as mean ± standard deviation, n (%), or median (interquartile range)

*BMI* Body mass index, *GFR* Glomerular filtration rate, *IABP* Intra-aortic balloon pump, *LAD* Left anterior descending coronary artery, *LCX* Left circumflex artery, *LVEF* Left ventricular ejection fraction, *MR* Mitral regurgitation, *NYHA* New York Heart Association, *RCA* Right coronary artery

MACE occurred in 32 patients (heart failure in 16, ischemic stroke in 16) during hospitalization or within 30 days postoperatively. The cases of stroke included one hemorrhagic stroke on postoperative day 2 and one case of common carotid artery occlusion on postoperative day 9, which resulted in death on postoperative day 28. None of the patients developed angina pectoris, myocardial infarction, or other cardiovascular events.

### Impact of patient and surgical characteristics on postoperative MACE

Next, we compared the characteristics of patients who did and did not develop MACE. As shown in Table 2, we found that an NYHA classification of 3 or 4, preoperative beta blocker use, reduced LVEF, emergency surgery, and intra-aortic balloon pump use were significantly associated with postoperative MACE. The volumes of intravenous fluids, transfusion, and vasopressor doses were similar between the two groups (Supplementary Table 1).

**Table 2:** Impact of patient and surgical characteristics on development of postoperative MACE

	MACE		
	Absent (*n* = 476)	Present (*n* = 32)	*P* value
Age, ≥ 75 years	163 (34)	12 (38)	0.704
Sex, male	378 (79)	21 (66)	0.076
BMI, ≥ 25 kg/m^2^	147 (31)	12 (38)	0.436
Smoking history	294 (62)	19 (59)	0.852
NYHA class, III or IV	40 (8)	13 (41)	< 0.001
Hypertension	394 (83)	24 (75)	0.336
Dyslipidemia	380 (80)	23 (72)	0.267
Diabetes mellitus	285 (60)	22 (69)	0.356
Lung disease	33 (7)	2 (6)	> 0.999
Malignancy	11 (2)	1 (3)	0.546
Atrial fibrillation	40 (8)	3 (9)	0.745
ESRD	47 (10)	7 (22)	0.066
Stroke	98 (21)	5 (16)	0.651
Preoperative beta blocker use	300 (63)	14 (44)	0.038
Preoperative statin use	331 (70)	19 (59)	0.240
Hemoglobin, < 10 g/dL	104 (22)	9 (28)	0.387
LVEF, < 40%	38 (8)	6 (19)	0.048
Moderate/severe MR	13 (3)	2 (6)	0.242
Emergency surgery	13 (3)	7 (22)	< 0.001
Presence of anastomosis LCX	366 (77)	23 (72)	0.520
Operative time, ≥ 4 h	117 (25)	9 (28)	0.674
IABP use	199 (42)	22 (69)	0.005
Blood loss, > 2000 mL	22 (5)	2 (6)	0.657

Data are shown as n (%)

*BMI* Body mass index, *ESRD* End-stage renal disease, *IABP* Intra-aortic balloon pump, *LCX* Left circumflex artery, *LVEF* Left ventricular ejection fraction, *MACE* Major adverse cardiovascular events, *MR* Mitral regurgitation, *NYHA* New York Heart Association

### Impact of intra-anesthetic vital signs on postoperative MACE

**Fig. 2:**
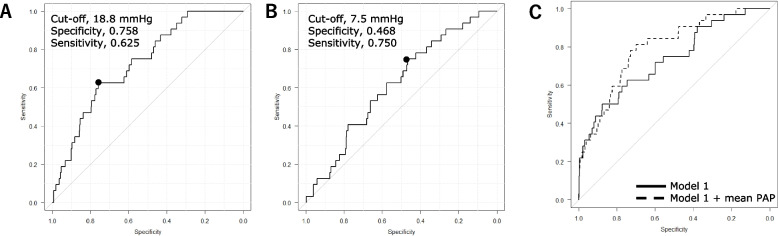
ROC curve analyses for the development of postoperative MACE. The mean PAP (**A**) and mean CVP (**B**) predicted development of postoperative MACE with moderate (AUC 0.726; 95% CI, 0.645 to 0.808) and low accuracy (AUC 0.616; 95% CI, 0.523 to 0.708), respectively. **C**. A multivariate ROC curve analysis was performed using mean PAP and model 1 (NYHA class, preoperative beta-blocker use, left ventricular ejection fraction, emergency surgery, intra-aortic balloon pump use, mean CVP). The solid line shows model 1, while the dashed line shows the addition of mean PAP to model 1. A multivariate ROC curve analysis showed that adding the mean PAP [mmHg] to model 1 achieved a better predictive value than model 1 alone in predicting MACE, with an AUC of 0.794, which is an increase of 0.059 (*P* = 0.036). The continuous net reclassification improvement and integrated discrimination improvement were 0.510 (95% CI, 0.169 to 0.851; *P* = 0.003) and 0.0071 (95% CI, -0.020 to 0.034; *P* = 0.603), respectively. *AUC* Area under the receiver operating characteristic curve, *CI* Confidence interval, *CVP* Central venous pressure, *MACE* Major adverse cardiovascular events, *NYHA* New York Heart Association, *PAP* Pulmonary artery pressure, *ROC* Receiver operating characteristic

To determine the effect of vital signs on the development of MACE, we compared the mean and coefficient of variation of each parameter after the induction of anesthesia between patients who did and did not develop MACE. As shown in Table 3, the mean intra-anesthetic PAP was significantly higher in patients with than without MACE (absolute difference, 2.6 mmHg; 95% confidence interval [CI], 1.5 to 3.8). The area under the ROC curve (AUC) of the mean intra-anesthetic PAP for the prediction of MACE was 0.726 (95% CI, 0.645 to 0.808). The optimal mean PAP cut-off was 18.8 mmHg, with a specificity of 75.8% and a sensitivity of 62.5% for predicting MACE (Fig. 2A). The mean CVP was also significantly higher in patients with than without MACE. The AUC of the mean intra-anesthetic CVP for the prediction of MACE was 0.616 (95% CI, 0.523 to 0.708). The optimal mean CVP cut-off was 7.5 mmHg, with a specificity of 46.8% and a sensitivity of 75.0% for predicting MACE (Fig. 2B).

**Table 3:** Impact of vital signs on development of postoperative MACE

		MACE		
		Absent (n = 476)	Present (n = 32)	*P* value
IBP, mean	mmHg	63.7 ± 6.1	62.3 ± 6.5	0.212
IBP, cov	%	23.9 ± 6.8	23.9 ± 6.3	0.987
HR, mean	/min	65.3 ± 8.8	66.8 ± 11.4	0.346
HR, cov	%	11.8 ± 3.9	13.5 ± 6.0	0.193
PAP, mean	mmHg	16.7 ± 3.4	19.3 ± 3.0	< 0.001
PAP, cov	%	18.4 ± 5.8	18.2 ± 6.9	0.854
CVP, mean	mmHg	7.6 ± 2.7	8.7 ± 2.3	0.020
CVP, cov	%	39.1 ± 29.5	32.1 ± 13.4	0.182
SpO_2_, mean	%	98.5 ± 1.2	98.2 ± 1.2	0.146
SpO_2_, cov	%	1.4 ± 1.1	1.5 ± 0.7	0.537

Data were shown in average ± standard deviation

*cov* Coefficient of variation, *CVP* Central venous pressure, *HR* Heart rate, *IBP* Invasive blood pressure, *MACE* Major adverse cardiovascular events, *PAP* Pulmonary artery pressure

A post hoc analysis found that the AUCs of the mean PAP for predicting heart failure and ischemic stroke were 0.731 (95% CI, 0.606 to 0.855) and 0.707 (95% CI, 0.602 to 0.812), respectively (Supplementary Figs. 1 and 2). However, the AUC of the mean CVP did not predict the development of heart failure and ischemic stroke.

### Impact of PAP on postoperative MACE

Multivariate logistic analysis of the incidence of MACE with a mean PAP of > 18.8 mmHg, a mean CVP of > 7.5 mmHg, and significant baseline characteristics in Table 2 as explanatory variables showed that PAP (odds ratio [OR], 3.77; 95% CI, 1.52 to 9.35) and an NYHA classification of 3 or 4 (OR, 3.50; 95% CI, 1.32 to 9.26) were significantly associated with the development of MACE (Table 4). The presence of mitral regurgitation (MR), anastomoses in the left circumflex artery (LCX), and end-stage renal disease remained potential contributors to pulmonary congestion [13]. However, these characteristics did not yield a P value of < 0.05 in Table 2. A sensitivity analysis that added these factors to model 1 found that a mean PAP of > 18.8 mmHg was an independent risk factor for MACE development (Supplementary Table 2).

**Table 4:** Logistic regression analysis on the development of MACE

	Univariate	Multivariate	
Variables	Odds ratio (95% CI)	Odds ratio (95% CI)	*P* value
NYHA class, III or IV	7.46 (3.43 to 16.2)	3.50 (1.32 to 9.27)	0.018
Preoperative beta blocker use	0.46 (0.22 to 0.94)	0.56 (0.25 to 1.23)	0.148
LVEF, < 40%	2.66 (1.03 to 6.86)	0.94 (0.29 to 3.00)	0.914
Emergency surgery	9.97 (3.66 to 27.2)	2.06 (0.60 to 7.01)	0.248
IABP use	3.05 (1.41 to 6.59)	1.61 (0.69 to 3.80)	0.273
Mean PAP, > 18.8 mmHg	5.17 (2.45 to 10.9)	3.78 (1.53 to 9.37)	0.004
Mean CVP, > 7.5 mmHg	2.27 (1.03 to 5.01)	1.02 (0.39 to 2.66)	0.970

*CI* Confidence interval, *CVP* Central venous pressure, *IABP* Intra-aortic balloon pump, *LVEF* Left ventricular ejection fraction, *NYHA* New York Heart Association, *MACE* Major adverse cardiovascular events, *PAP* Pulmonary artery pressure

A multivariate ROC curve analysis showed that adding the mean PAP [mmHg] to model 1 (NYHA class, preoperative beta blocker use, LVEF [%], emergency surgery, intra-aortic balloon pump use, mean CVP [mmHg]) achieved a better predictive value than model 1 alone in predicting MACE, with an AUC of 0.794, which is an increase of 0.059 (*P* = 0.036) (Fig. 2C). Furthermore, a multivariate ROC analysis was performed for model 1 with the addition of the presence of MR, the number of anastomoses in the LCX, and the estimated glomerular filtration rate (mL/min/1.73m^2^) (model 2). Supplementary Fig. 3 shows a significant increase in the AUC when pulmonary artery pressure was added to model 2 compared with model 2 alone (0.814 versus 0.760, *P* = 0.028).

### Impact of pre-incision and post-incision PAP on MACE

We could not investigate which surgical manipulations were associated with elevated PAP because the data on PAP did not correspond to the surgical manipulations. Instead, we investigated the PAP before and after the start of the surgical procedure to determine whether the high PAP was due to the surgery. A 2 × 2 contingency table was prepared before and after the surgical incision and with and without MACE (Table 5). The results showed that the PAP was significantly higher in the MACE group before and after the skin incision than in the group without MACE (Table 5). These observations suggest that preoperative PAP elevation is associated with the development of MACE.

**Table 5:** Effect of pre- and post-incision PAP on MACE

		MACE		
		Absent (*n* = 476)	Present (*n* = 32)	*P* value
PAP				
Pre-incision	mmHg	15.4 ± 3.8	18.3 ± 4.9	< 0.001
Post-incision	mmHg	16.9 ± 3.4	19.6 ± 2.9	< 0.001

Data are shown as mean ± standard deviation

*MACE* Major adverse cardiovascular events, *PAP* Pulmonary artery pressure

### Time-series clustering of mean PAP

**Fig. 3:**
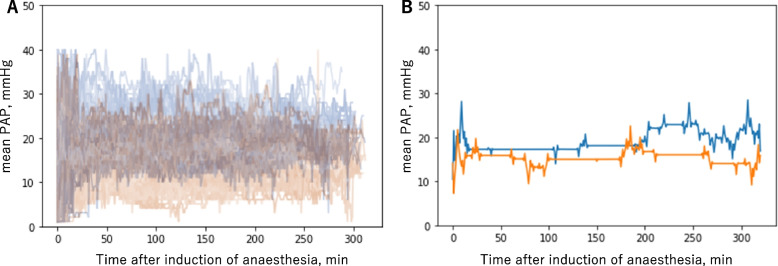
Time-series clustering with artificial intelligence for PAP. We applied time-series clustering to statistically significant variables using Python 3.9.0 and tslearn 0.5.2 (https://tslearn.readthedocs.io/en/stable/). The whole time-series changes in (**A**) PAP and (**B**) the barycentres are shown. The artificial intelligence divided the patients into a high PAP group (blue line, *n* = 197) and low PAP group (red line, *n* = 311), with a silhouette score of 0.7066. *PAP* Pulmonary artery pressure

As a sensitivity analysis, we applied time-series clustering to the mean PAP using artificial intelligence. As shown in Fig. 3, artificial intelligence divided all samples into a high PAP group (*n* = 197) and a low PAP group (*n* = 311), with a silhouette score of 0.7066. Twenty-two (11.2%) and 10 (3.2%) patients developed MACE in the high and low PAP groups, respectively, indicating a significantly higher percentage of MACE in the high PAP group (*P* = 0.0005; OR, 3.77; 95% CI, 1.67 to 9.14).

## Discussion

In this retrospective study, we analyzed the association between intraoperative vital sign variability and postoperative complications after OPCAB surgery. Thirty-two patients in this study developed postoperative MACE. The mean PAP was significantly higher in patients with than without MACE. ROC analysis showed that the mean PAP could predict the occurrence of MACE after OPCAB with moderate accuracy, with an optimal cut-off PAP value of 18.8 mmHg. Time-series clustering as a sensitivity analysis also showed a significantly higher incidence of MACE in the high than low PAP group.

Cardiogenic pulmonary edema is most often a result of LV systolic and/or diastolic impairment [14]. Valve abnormalities, such as MR, also contribute to the development of pulmonary congestion, and cardiac positioning during LCX anastomosis in OPCAB surgery often causes a transient MR [13]. In the present study, high PAP remained predictive of the development of postoperative complications after adjusting for LV systolic function, renal function, MR, and the number of LCX anastomoses (Supplementary Table 2 and Supplementary Fig. 1). However, the present study could not evaluate LV diastolic function due to a lack of available data. Patients with pulmonary hypertension are classified into five groups based upon etiology and mechanism: group 1 (pulmonary arterial hypertension), group 2 (due to left-sided heart disease), group 3 (due to chronic lung disorders and hypoxemia), group 4 (due to pulmonary artery obstructions), and group 5 (due to unknown mechanisms) [15]. The most common etiology of pulmonary hypertension is secondary to left-sided heart disease (group 2) [14]. Furthermore, a meta-analysis showed that preoperative diastolic dysfunction is an independent risk factor for postoperative heart failure [16]. Therefore, the lack of data regarding diastolic function is one of the important limitations of the present study. However, because there are many different methods and cut-offs for diagnosing diastolic dysfunction [16, 17], studies are warranted to determine the optimal method of assessing LV diastolic function for predicting the development of postoperative complications.

Pulmonary hypertension, defined as a mean PAP of ≥ 25 mmHg at rest, is associated with stroke development [18]. According to a database analysis, pulmonary hypertension is a risk factor for stroke development after non-cardiac surgery [19]. However, the present study included no patients with confirmed or suspected preoperative pulmonary hypertension (Table 1). One research group recently proposed defining pulmonary hypertension as more than 20 mmHg, which is twice the standard deviation from the mean PAP in healthy individuals [20]. Furthermore, two large cohort studies revealed that borderline pulmonary hypertension with a mean PAP of > 19 mmHg is associated with death and hospitalization [21, 22]. Thus, evidence is accumulating that high pulmonary hypertension within a range that does not meet the definition of typical pulmonary hypertension is a cardiovascular event. As spontaneous and mechanical ventilation reportedly result in differences in pulmonary arterial pressure [23], our results cannot be directly extrapolated to conditions under spontaneous ventilation. However, because our findings are the first evidence that intraoperative borderline pulmonary hypertension may affect the prognosis of patients undergoing coronary artery bypass grafting, the validity of these findings requires verification in future prospective, large-scale studies.

The mechanism underlying the association between high PAP and the development of ischemic stroke is unknown. Some observational studies have demonstrated an association between pulmonary hypertension and ischemic stroke but have not provided reasonable explanations for this association [18, 24]. The association between pulmonary hypertension and ischemic stroke occurs in certain pathological conditions, such as sickle cell disease [25], hereditary hemorrhagic telangiectasia [26], HIV infection [27], and methamphetamine use [28]. However, these conditions are rare in Japan, and the patients in our cohort did not have these diseases. The present study revealed that borderline pulmonary hypertension influences the development of postoperative complications. Therefore, accumulating PAP data may provide insights into the association between pulmonary hypertension and postoperative cerebral infarction.

In non-cardiac surgery, PAC insertion should be considered only in select patients with hemodynamic instability [29]. The use of PACs in non-cardiac surgery has markedly declined because of non-negligible fatal complications [30]; similarly, in coronary artery bypass grafting, the use of PACs is reportedly neither beneficial nor associated with increased mortality and has a higher risk of severe end-organ complications [31, 32]. Importantly, however, no studies have provided a standardized method to determine the specific data that should be retrieved from the PAC to make decisions. PAC insertion is still frequently performed in OPCAB [33], which may mean that cardiac surgeons and anesthesiologists are empirically aware of the importance of PACs. The present study showed that a PAP cut-off of 18.8 mmHg under general anesthesia is moderately predictive of the development of MACE. A clear target PAP cut-off may enable the accurate prediction of complications. Although waveforms are now widely analyzed using time-series clustering, such as for electroencephalography [34] and electrocardiography [35], no previous reports have described the use of time-series clustering for detecting a trend in the mean PAP during cardiac surgery (Fig. 3). In addition, the prediction of complications may improve if absolute values or waveforms can be analyzed mechanically using artificial intelligence.

A recent study reported no difference in early-term outcomes after OPCAB surgery between pulmonary hypertension and non-hypertension groups [36]. However, Table 2 suggests that preoperative NYHA, beta blocker use, and emergency surgery are important confounding factors in the development of postoperative MACE. The difference in analysis methods between studies may account for the difference in our results regarding the impact of borderline pulmonary hypertension on the prognosis after OPCAB surgery.

The present study had several limitations. First, we did not assess LV diastolic function or other indicators of cardiac function, such as the pulmonary artery wedge pressure and preoperative B-type natriuretic peptide concentration. Second, this study did not clarify when elevated PAP was associated with prognosis. Third, this was an observational study based on a review of medical records. Therefore, the exact causal relationship between PAP elevation and the development of ischemic stroke is unknown. Fourth, other data obtained from the PAC, such as cardiac output and mixed venous oxyhemoglobin saturation, were not analyzed because they were not recorded as continuous electronic data. Sixth, the depth of anesthesia [37] and ventilatory conditions, such as airway pressure, were not assessed. Seventh, this was a single-center analysis that included a small number of events, although the patient characteristics shown in Table 1 were comparable to those in a previous Japanese database study [38]. Eighth, although our definition of MACE included a wide range of diseases, only heart failure and ischemic stroke occurred in the present cohort. Each event was shown to be associated with borderline pulmonary hypertension (Supplementary Figs. 1 and 2), but the relationship between PAP and each pathology warrants further investigation.

## Conclusion

In this retrospective study of OPCAB surgery, a significant association was found between elevated mean PAP, even if the PAP did not meet diagnostic criteria for pulmonary hypertension, and the development of postoperative MACE. This study provided a PAP cut-off value of 18.8 mmHg in anesthetized patients for the prediction of postoperative MACE. Future prospective studies are needed to clarify the clinical significance of intraoperative borderline pulmonary hypertension.

## Supplementary Information

**Additional file 1.**

## Data Availability

The datasets used and/or analyzed during the current study are available from the corresponding author on reasonable request.

## Abbreviations

AUC: Area under the receiver operating characteristic curve
CI: Confidence interval
CVP: Central venous pressure
IQR: Interquartile range
LCX: Left circumflex artery
LV: Left ventricular
LVEF: Left ventricular ejection fraction
MACE: Major adverse cardiovascular events
MR: Mitral regurgitation
NYHA: New York Heart Association
OPCAB: Off-pump coronary artery bypass
OR: Odds ratio
PAC: Pulmonary artery catheter
PAP: Pulmonary artery pressure
ROC: Receiver operating characteristic
