# Supplementary material for: Association between intraoperative pulmonary artery pressure and cardiovascular complications after off-pump coronary artery bypass surgery: a single-center observational study
Source: BMC Anesthesiol. 2023 Apr 6;23:114. doi: 10.1186/s12871-023-02057-5 (PMC10077666; doi:10.1186/s12871-023-02057-5)
Supplement: Supplementary file 1 — Additional file 1. [file 12871_2023_2057_MOESM1_ESM.docx]

Supplementary Table 1. Impact of intraoperative drug use on development of postoperative MACE

|  |  | MACE | |  |
| --- | --- | --- | --- | --- |
|  |  | Absent (n = 476) | Present (n = 32) | P value |
| Crystalloid solutions | mL | 2650 (2138, 3328) | 2490 (2050, 3025) | 0.40 |
| Colloid solutions | mL | 500 (0, 1000) | 0 (0, 1000) | 0.23 |
| Red blood cell | mL | 0 (0, 110) | 0 (0, 350) | 0.15 |
| Fresh frozen plasma | mL | 0 (0, 0) | 0 (0, 0) | 0.80 |
| Albumin | g | 12.5 (0, 25) | 13.75 (12.5, 28.1) | 0.077 |
| Ephedrine | mg | 0 (0, 0) | 0 (0, 0) | 0.88 |
| Phenylephrine | mg | 0.8 (0.5, 1.3) | 0.7 (0.3, 1.4) | 0.26 |
| Nicardipine | mg | 0 (0, 0) | 0 (0, 0) | 0.082 |
| Fentanyl | mg | 1.3 (1.0, 1.6) | 1.3 (1.0, 1.4) | 0.15 |

Data were shown in median (interquartile range). Statistical comparisons were performed using the Mann–Whitney U test.

MACE, major adverse cardiovascular events.

Supplementary Table 2. Logistic regression analysis on the development of MACE

|  | Multivariate | |
| --- | --- | --- |
| Variables | Odds ratio (95% CI) | P value |
| NYHA class, III or IV | 3.36 (1.24 to 9.07) | 0.017 |
| Preoperative beta blocker use | 0.61 (0.272 to 1.37) | 0.23 |
| LVEF, <40% | 1.01 (0.32 to 3.20) | 0.99 |
| Emergency surgery | 2.04 (0.60 to 7.00) | 0.26 |
| IABP use | 1.61 (0.68 to 3.80) | 0.28 |
| Mean PAP, >18.8 mmHg | 3.74 (1.48 to 9.45) | 0.0053 |
| Mean CVP, >7.5 mmHg | 0.92 (0.35 to 2.47) | 0.88 |
| ESRD | 2.21 (0.80 to 6.07) | 0.13 |
| Moderate/severe MR | 0.73 (0.13 to 4.18) | 0.72 |
| Number of LCX anastomoses | 0.72 (0.34 to 1.5) | 0.38 |

CI, confidence interval; CVP, central venous pressure; ESRD, end-stage renal disease; IABP, intra-aortic balloon pump; LVEF, left ventricular ejection fraction; NYHA, New York Heart Association; MACE, major adverse cardiovascular events; MR, mitral regurgitation; PAP, pulmonary artery pressure.

Supplementary Figure 1

The area under the ROC curve of the mean intra-anesthetic PAP for the prediction of heart failure was 0.731 (95% CI, 0.606 to 0.855). The optimal mean PAP cut-off was 19.0 mmHg, with a specificity of 76.0% and a sensitivity of 62.5% for predicting heart failure.

Supplementary Figure 2

The area under the ROC curve of the mean intra-anesthetic PAP for the prediction of ischemic stroke was 0.707 (95% CI, 0.602 to 0.812). The optimal mean PAP cut-off was 18.8 mmHg, with a specificity of 74.6% and a sensitivity of 62.5% for predicting ischemic stroke.

Supplementary Figure 3


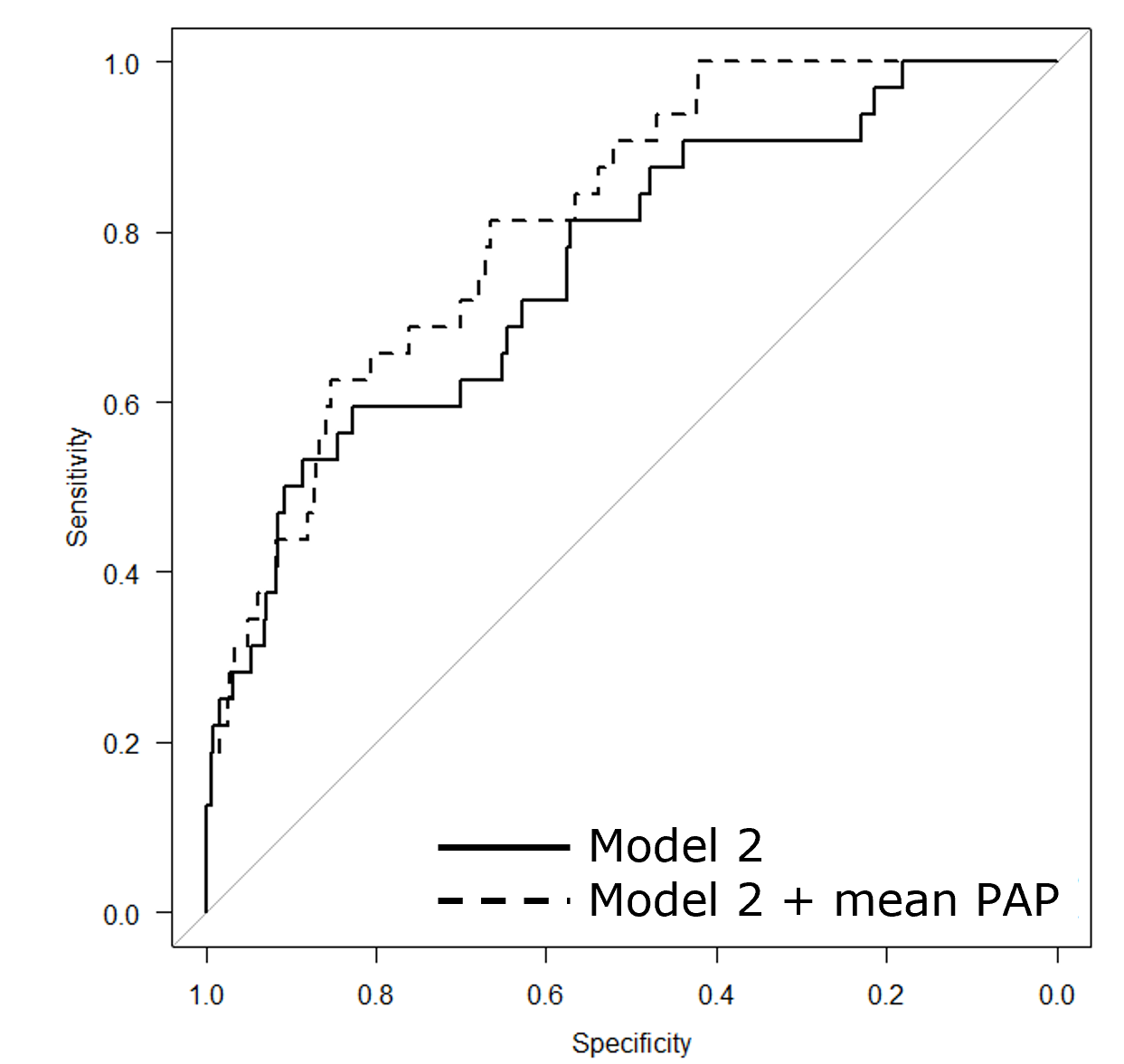


Multivariate ROC curve analyses were performed using mean PAP (mmHg) with or without model 2 (model 1, presence of mitral regurgitation, the number of anastomoses in the left circumflex artery, estimated glomerular filtration rate). A solid line showed model 2, and a dashed line indicated adding mean PAP (mmHg) to model 2. The continuous net reclassification improvement and integrated discrimination improvement were 0.477 (95% CI, 0.130 to 0.8241; P = 0.007) and 0.003 (95% CI, -0.021 to 0.028; P = 0.790), respectively.
